# Rationale and design of LUX-Head & Neck 1: a randomised, Phase III trial of afatinib versus methotrexate in patients with recurrent and/or metastatic head and neck squamous cell carcinoma who progressed after platinum-based therapy

**DOI:** 10.1186/1471-2407-14-473

**Published:** 2014-06-28

**Authors:** Jean-Pascal H Machiels, Lisa F Licitra, Robert I Haddad, Makoto Tahara, Ezra EW Cohen

**Affiliations:** 1 Cancer Center, Service d’Oncologie Médicale, Cliniques Universitaires Saint-Luc and Institut de Recherche Clinique et Expérimentale (Pole MIRO), Université Catholique de Louvain, Brussels, Belgium; 2 Istituto Nazionale Tumori, Milan, Italy; 3 Dana-Farber Cancer Institute/Harvard Medical School, Boston, MA, USA; 4 National Cancer Center Hospital East, Kashiwa, Japan; 5 University of California San Diego Moores Cancer Center, La Jolla, CA, USA

**Keywords:** Afatinib, Methotrexate, Head and neck, Phase III, Recurrent, Metastatic

## Abstract

**Background:**

Patients with recurrent and/or metastatic (R/M) head and neck squamous cell carcinoma (HNSCC) receiving platinum-based chemotherapy as their first-line treatment have a dismal prognosis, with a median overall survival (OS) of ~7 months. Methotrexate is sometimes used following platinum failure or in patients not fit enough for platinum therapy, but this agent has not demonstrated any OS improvement. Targeted therapies are a novel approach, with the EGFR-targeting monoclonal antibody cetuximab (plus platinum-based chemotherapy) approved in the US and Europe in the first-line R/M setting, and as monotherapy following platinum failure in the US. However, there is still a high unmet medical need for new treatments that improve outcomes in the second-line R/M setting following failure on first-line platinum-containing regimens. Afatinib, an irreversible ErbB family blocker, was recently approved for the first-line treatment of EGFR mutation-positive metastatic non-small cell lung cancer. Afatinib has also shown clinical activity similar to cetuximab in a Phase II proof-of-concept HNSCC trial. Based on these observations, the Phase III, LUX-Head & Neck 1 study is evaluating afatinib versus methotrexate in R/M HNSCC patients following progression on platinum-based chemotherapy in the R/M setting.

**Methods/Design:**

Patients with progressive disease after one first-line platinum-based chemotherapy are randomised 2:1 to oral afatinib (starting dose 40 mg once daily) or IV methotrexate (starting dose 40 mg/m^2^ once weekly) administered as monotherapy with best supportive care until progression or intolerable adverse events. Efficacy of afatinib versus methotrexate will be assessed in terms of progression-free survival (primary endpoint). Disease progression will be evaluated according to RECIST v1.1 by investigator and independent central review. Secondary endpoints include OS, tumour response and safety. Health-related quality of life and biomarker assessments will also be performed.

**Discussion:**

If the LUX-Head & Neck 1 trial meets its primary endpoint, it will demonstrate the ability of afatinib to elicit an improved treatment benefit versus a commonly used chemotherapy agent in the second-line treatment of R/M HNSCC patients who have failed on first-line platinum-based therapy, confirm the clinical efficacy of afatinib observed in the Phase II proof-of-concept study, and establish a new standard of care for this patient population.

## Background

Head and neck squamous cell carcinoma (HNSCC) has an incidence of more than 600,000 new cases worldwide per year [1]. The majority of HNSCC patients are diagnosed in the later stages of the disease, with more than half of patients having locoregionally advanced (LA) HNSCC at the time of diagnosis and approximately 10% of patients having metastatic disease [2]. Prognosis in LA patients is poor, with around 50% of unresectable patients relapsing 5 years after receiving definitive chemoradiotherapy (CRT) [3,4], a standard treatment in this setting. Resectable patients receiving adjuvant CRT following surgery have a 5-year recurrence rate of 20% [5]. Furthermore, recurrent and/or metastatic (R/M) HNSCC patients receiving first-line chemotherapy only have a median overall survival (OS) of approximately 7 months [6]. Therefore, the R/M setting represents a group of patients who require novel treatment approaches.

### Non-targeted treatments for R/M HNSCC

The most common non-targeted treatment approach in R/M HNSCC is a platinum-containing agent combined with either a taxane or 5-fluorouracil (5-FU) [7]. Response rates and OS in R/M HNSCC following platinum-based doublet chemotherapy in the first-line setting are low. Results from four large randomised studies in this setting comparing cisplatin plus 5-FU with other single-agent chemotherapy agents demonstrated that combination regimens elicited response rates of around 20–30% [8-11]. In three further studies assessing cisplatin in combination with paclitaxel, median OS was reported to be between 6.5 and 8.0 months [12-14]. Moreover, following failure on a platinum-containing regimen there is no defined standard of care and second-line treatment options for R/M patients are limited, thus highlighting the need for alternative treatments that can improve outcomes in these patients.

Methotrexate is commonly used in R/M HNSCC [7] and continues to be used as a standard comparator in some Phase III trials, mainly after platinum failure or in patients judged unfit for platinum therapy [9,15-17]. Taxanes have also been used in this setting. However, no study has been able to show that these agents improve OS. The ability of methotrexate to increase OS in HNSCC patients has not been formerly demonstrated in Phase III trials. This agent produces a response of short duration (approximately 3–6 months) in around 4–24% of cases and only rarely elicits complete responses (CRs) [15,16,18-20].

### Targeted treatment approaches

A recent approach to new cancer therapies has been to develop targeted agents that inhibit particular signalling pathways implicated in tumourigenesis. Epidermal growth factor receptor (EGFR; ErbB1) is a member of the ErbB family of receptor tyrosine kinases that plays an integral role in the oncogenesis of several ErbB-driven cancers, including HNSCC [21]. Overexpression of EGFR provides tumour cells with growth and survival advantages, and this process is thought to substantially contribute to the aggressive nature of cancer cell proliferation. Approximately 90% of patients with HNSCC overexpress EGFR and prognosis for these patients can be lower than for patients without high levels of EGFR expression, with increased EGFR expression correlating with a reduction in recurrence-free survival or OS rates [21]. One study has shown that in patients with laryngeal squamous cell carcinoma, those with low EGFR expression levels have a 5-year OS rate of 81% compared with 25% for patients with high levels of EGFR expression [22].

Cetuximab is an EGFR-targeting monoclonal antibody and is the only targeted treatment approved in the US and Europe for the treatment of HNSCC in combination with radiotherapy for LA disease and in combination with platinum-based chemotherapy for R/M disease [23,24]. It is also approved in the US as monotherapy in R/M HNSCC following progression on platinum-based chemotherapy [23]. In a Phase III trial in R/M patients, combination treatment with cetuximab and cisplatin led to an objective response rate (ORR) of 26% versus 10% with cisplatin plus placebo (p = 0.03) [25]. However, owing to this trial being underpowered, no significant difference was observed for progression-free survival (PFS) or OS in both arms. In the larger confirmatory EXTREME study, cetuximab in combination with platinum-based chemotherapy elicited an OS benefit in untreated R/M HNSCC patients versus chemotherapy alone [6]. The median OS was prolonged from 7.4 months in patients receiving chemotherapy alone to 10.1 months in the cetuximab plus chemotherapy arm. Median PFS was also increased from 3.3 months in the chemotherapy alone group to 5.6 months in the combination group.

In platinum-refractory R/M HNSCC patients with disease progression, three studies have been performed assessing the efficacy of cetuximab either alone or in combination with platinum-based chemotherapy. In 2005, two trials evaluated cetuximab in combination with either cisplatin or carboplatin in this setting. Herbst *et al.* reported an ORR of 10% and OS of 5.2 months in patients receiving cetuximab plus cisplatin [26] and similar results were observed by Baselga *et al.* who determined an ORR of 10% and OS of 6 months following treatment with cetuximab plus cisplatin or carboplatin [27]. Cetuximab has also been investigated as a monotherapy in the R/M population in patients who have failed platinum-based chemotherapy, with a best overall response rate of 13% and OS of 5.9 months observed [28]. This trial suggests that single-agent cetuximab offers similar efficacy to combination treatment with platinum-based chemotherapy in R/M HNSCC patients refractory to platinum-containing therapy. A pooled analysis of these three trials was performed in 2008, which compared them to a retrospective trial by Leon *et al.*[29]. Leon *et al.* assessed the outcomes of platinum-refractory R/M HNSCC patients treated between 1990 and 2000 with best supportive care or various second-line therapies. This indirect comparison indicated that median OS may be increased by approximately 2 months when cetuximab is administered following platinum failure, with OS ranging between 5.2 and 6.1 months in the cetuximab studies versus 3.4 and 3.6 months in Leon *et al.*’s retrospective analysis [30].

Several other targeted agents are currently being investigated for HNSCC, including the monoclonal antibody panitumumab, the small-molecule tyrosine kinase inhibitors dacomitinib and lapatinib, and the oncolytic virus reolysin. The monoclonal antibody nimotuzumab is already approved in numerous countries, including Brazil, India, China, Argentina and Indonesia. However, this is still under investigation for HNSCC in the US and Europe.

Acquired or primary resistance to targeted therapies is common, with several mechanisms being implicated in this process. These postulated or hypothetical mechanisms include receptor-independent activation of downstream signalling cascades, cross-talk with other receptor tyrosine kinases, and environmental factors, such as viral infections and inflammatory agents [31]. A novel approach to overcome treatment resistance is inhibition of multiple ErbB family members simultaneously or binding multiple ErbB family members irreversibly [32]. By blocking all ErbB family members, greater efficacy may be achieved as all ErbB-driven oncogenic pathways are compromised. Furthermore, irreversible inhibition, mediated by covalent binding to specific residues of the target, may lead to sustained suppression of tumour growth as prolonged cellular activity is inhibited.

### Afatinib, an irreversible ErbB family blocker

Afatinib is an oral ErbB family blocker that completely and irreversibly blocks signalling by all relevant ErbB family members, including EGFR, human epidermal growth factor receptor-2 (ErbB2) and ErbB4, and also blocks transphosphorylation of ErbB3 [33,34]. It is approved in the US for the first-line treatment of EGFR mutation-positive metastatic NSCLC and it is also being developed for the treatment of a number of other ErbB-driven tumours, including breast cancer and HNSCC.

In the LUX-Lung clinical trial programme, afatinib has been investigated for the treatment of EGFR mutation-positive NSCLC either in the first-line setting [35-37] or in patients with no more than one prior chemotherapy [38]. It has also been assessed following chemotherapy and/or EGFR tyrosine kinase inhibitor therapy [39-43]. In the proof-of-concept LUX-Lung 2 trial, afatinib monotherapy elicited an ORR of 61% in NSCLC patients [38] and in LUX-Lung 3, to our knowledge being the largest, prospective, randomised trial in EGFR mutation-positive NSCLC patients, the primary endpoint of PFS was met, with a median PFS of 11.1 months observed for afatinib-treated patients versus 6.9 months in chemotherapy-treated patients [35]. Afatinib has also demonstrated a manageable safety profile, with recent pooled data analyses in patients with solid tumours showing that gastrointestinal and dermatological adverse events in particular can be effectively managed in this patient population [44,45].

In HNSCC, afatinib has demonstrated preclinical activity in both *in vitro* and *in vivo* models [46,47] and clinical activity in a proof-of-concept Phase II study [48,49]. In the human HNSCC FaDu cell line, afatinib inhibited tumour cell proliferation in the low nanomolar concentration range, with additive growth inhibitory effects demonstrated when combined with standard chemotherapies versus single-agent treatment [47]. The Phase II proof-of-concept study showed comparable activity between afatinib and cetuximab in R/M HNSCC patients following failure of platinum-based chemotherapy. In Stage I, ORRs were 8.1% in afatinib-treated patients and 9.7% in cetuximab-treated patients (independent central review) [48]. Furthermore, in Stage II of the study, after crossover to the opposite treatment arm, afatinib elicited a disease control rate of 33% in patients who received cetuximab in Stage I (vs. 19% in cetuximab-treated patients after crossover from afatinib) and demonstrated PFS of 9.3 weeks in the afatinib group versus 5.7 weeks in the cetuximab group, suggesting sequential therapy with afatinib may be efficacious in patients pretreated with an EGFR-targeted therapy [49]. Therefore, these data warrant further investigation of this compound for the treatment of R/M HNSCC.

In afatinib monotherapy trials, the maximum tolerated dose was determined to be continuous daily afatinib at either 40 mg or 50 mg [50,51]. Afatinib 50 mg/day was the starting dose used in the proof-of-concept LUX-Lung 2 trial in EGFR mutation-positive patients who had received no more than one previous chemotherapy [38]. However, the dose was reduced to 40 mg/day to improve the safety profile of afatinib and, as there was no difference in efficacy in patients receiving both doses, 40 mg/day afatinib was the starting dose used in the subsequent LUX-Lung 3 and 6 trials [35,36]. In the HNSCC proof-of-concept trial, a starting dose of 50 mg/day afatinib was used [48]; however, afatinib demonstrated a more manageable safety profile at 40 mg/day in this study and so this is the chosen starting dose of afatinib in LUX-Head & Neck 1, with individual dosing allowed depending on how well patients tolerate treatment. It has been established that using a dose-reduction scheme in the administration of afatinib is an effective approach to minimising the consequences of adverse events and discontinuation of afatinib. Therefore, this is the approach being adopted in LUX-Head & Neck 1.

The LUX-Head & Neck 1 study (NCT01345682) has been initiated to assess the efficacy and safety of afatinib versus methotrexate in the second-line treatment of R/M HNSCC patients following failure of first-line platinum-based chemotherapy. Given methotrexate is a standard treatment in R/M HNSCC in many countries, and is used as a standard comparator in other Phase III trials, this agent is considered an appropriate comparator in this study. In particular, this second-line trial is powered to detect superiority of afatinib over methotrexate in terms of a PFS and OS benefit. There are currently no approved predictive tumour- or serum-derived biomarkers guiding treatment with ErbB-directed therapies in HNSCC. Therefore, this study also includes a biomarker assessment part.

## Methods/Design

### Objectives

The primary objective of LUX-Head & Neck 1 is to evaluate the superiority of afatinib to methotrexate in terms of PFS in patients with R/M HNSCC who have progressed after platinum-based therapy for R/M disease. Progression-free survival has been chosen as the primary endpoint of this study because further treatments following disease progression potentially dilute the effect on survival afforded by the treatments under investigation. Secondary objectives include OS, ORR, health-related quality of life (HRQoL) and safety of afatinib versus methotrexate in this patient population.

### Study design and treatments

**Figure 1:**
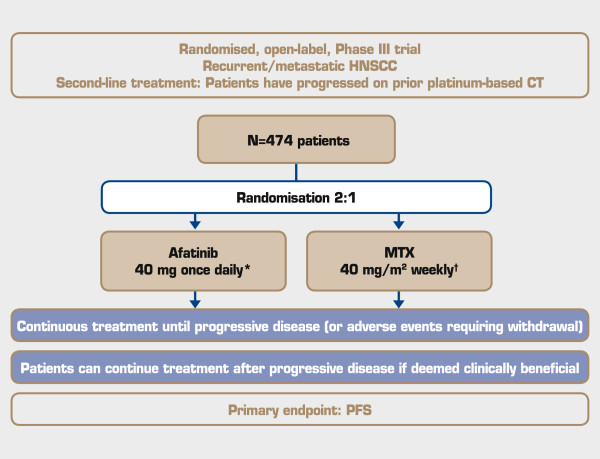
**Trial design.** *Dose escalation to 50 mg once daily and/or reduction to 40, 30, then 20 mg once daily. ^†^Dose can be escalated to 50 mg/m^2^ weekly and/or reduction to 40, 30, then 20 mg/m^2^ weekly. HNSCC = head and neck squamous cell carcinoma; CT = chemotherapy; MTX = methotrexate; PFS = progression-free survival.

In this Phase III, open-label, multicentre, randomised trial, eligible patients will be randomised 2:1 to continuous, once-daily afatinib or weekly methotrexate, administered as monotherapy with best supportive care. Randomisation will be stratified based on Eastern Cooperative Oncology Group (ECOG) performance score (0 vs. 1) and prior use of EGFR-targeted antibody therapy in the R/M setting. Randomised patients will be treated until progression, unacceptable adverse events (AEs) or other reasons necessitating treatment withdrawal (Figure 1). Patients may continue study medication beyond disease progression in case of clinical benefit, as long as this is judged beneficial by the investigator.

Individualised dosing will be adopted based on tolerability, with the afatinib starting dose being 40 mg once daily, increasing to 50 mg following minimal drug-related AEs after at least 4 weeks of treatment. The afatinib dose will be reduced in decrements of 10 mg to a minimum of 20 mg in the event of specific drug-related AEs. Methotrexate will be administered as intravenous bolus injections of 40 mg/m^2^ once a week, with the option to increase the dose to 50 mg/m^2^ in the event of no or minimal drug-related AEs after at least 2 weeks of treatment. The methotrexate dose will be reduced in decrements of 10 mg/m^2^ to a minimum of 20 mg/m^2^ in the event of drug-related AEs.

LUX-Head & Neck 1 is being conducted worldwide and target accrual is 474 patients. The trial is being carried out in compliance with the protocol, the principles laid down in the Declaration of Helsinki, in accordance with the International Conference on Harmonization Guideline for Good Clinical Practice (ICH-GCP) and in accordance with applicable regional-specific regulatory requirements. The study protocol has been reviewed by Independent Ethics Committees in each country, according to national and international regulations, and written informed consent will be obtained from each patient before any study-specific screening assessments are performed, according to ICH-GCP and regulatory and legal requirements of the participating country. The Independent Ethics Committees that reviewed the study protocol are as follows: Argentina (Comité de Etica de Investigacion Instituto de Oncología Angel Roffo; Cte de Docencia e Investigación de ISIS; Comité Independiente de Etica para Ensayos en Farmacología Clinica; Comité de Docencia e Investigación de CETEN; Comité de Etica del CER Investigaciones Clinicas); Austria (Ethics Committee of the Medical University of Vienna); Belgium (Commissie Medische Ethiek – UZ Leuven); Brazil (Comitê de Ética em Pesquisa da Faculdade de Medicina da Universidade de São Paulo; Comitê de Ética em Pesquisa em Seres Humanos do Hospital Pró-Cardíaco Pronto Socorro Cardiológico; Comitê de Ética em Pesquisa em Seres Humanos da Irmandade da Santa Casa de Misericórdia de Porto Alegre; Comitê de Ética em Pesquisa em Seres Humanos da Fundação Pio XII - Hospital do Câncer de Barretos; Comitê de Ética em Pesquisa em Seres Humanos da Fundação Hospital Amaral Carvalho; Comitê de Ética em Pesquisa em Seres Humanos da Fundação Antônio Prudente - Hospital do Câncer - AC Camargo; Comitê de Ética em Pesquisa em Seres Humanos da Universidade de Passo Fundo); Czech Republic (Ethics Committee of Fakultní nemocnice Olomouc a Lékařské fakulty UP v Olomouci; Ethics Committee of Teaching Hospital Bulovka; Ethics Committee of General Teaching Hospital Prague); Denmark (De Videnskabsetiske Komiteer for Region Hovedstaden); France (Comité de Protection des Personnes Sud-Est IV, Centre Léon Bérard); Germany (Ethik-Kommission der Medizinischen Fakultät der Universität Duisburg-Essen; Ethik-Kommission des Landes Berlin – Landesamt für Gesundheit und Soziales; Ethik-Kommission an der Medizinischen Fakultät der RWTH Aachen; Ethik-Kommission an der Medizinischen Fakultät der Universität Leipzig; Ethikkommission der Ärztekammer Hamburg; Ethikkommission der Medizinischen Hochschule Hannover; Medizinische Ethikkommission II der Medizinischen Fakultät Mannheim; Geschäftsstelle der Ethik-Kommission; Ethikkommission an der Technischen Universität Dresden); Greece (National Ethics Committee); Israel (Helsinki Committee); Italy (Comitato Etico Interaziendale dell’asl s. croce e carle di Cuneo; Comitato Etico Regionale della Liguria; Comitato Etico dell’IRCCS Istituto Nazionale per lo Studio e la Cura dei Tumori Fondazione Giovanni Pascale di Napoli; Comitato Etico Interaziendale della Provincia di Messina; Comitato Etico Palermo; Comitato Etico per la Sperimentazione Clinica della Provincia di Venezia e IRCCS San Camillo; Comitato Etico Centrale IRCCS Lombardia – C/o Fondazione IRCCS Istituto Nazionale dei Tumori; Comitato Etico dell’AOU di Cagliari; Comitato Etico della Ausl della Valle d’Aosta; Comitato Etico Lazio 1 - Azienda Ospedaliera S. Camillo-Forlanini); Japan (IRB of Jichi Medical University Hospital; The IRB of National Cancer Center Hospital East; IRB of National Hospital Organization Tokyo Medical Center; IRB of Shizuoka Cancer Center; IRB of Aichi Cancer Center Hospital; IRB of Kobe University Hospital; IRB of National Hospital Organization Shikoku Cancer Center; IRB of Tokai University Hospital; IRB of Osaka Medical Center for Cancer and Cardiovascular Diseases; IRB of Hyogo Cancer Center; IRB of The Jikei University Hospital of Medicine; IRB of Japanese Foundation for Cancer Research; IRB of Miyagi Cancer Center); Mexico (Instituto Nacional de Cancerología – Comité de Bioética; Instituto Nacional de Cancerología – Comité Científico); Russia (Ethics Committee within Clinical Oncology Center; Ethics Committee within Bashkir State Medical University; Ethics Committee within Kursk Regional Clinical Oncological Center; Ethics Committee within St. Petersburg Pavlov State Medical University; Local Ethics Committee within Blokhin Cancer Research Center; Ethics Committee within Pyatigorsk Oncology Center); South Africa (Human Research Ethics Committee; Faculty of Health Sciences Research Ethics Committee – University of Pretoria and Pretoria Academic Hospitals; Pharma Ethics); Spain (CEIC Hospital Universitari de la Vall d’Hebrón; CEIC Área de Salud de Salamanca; Comité Etico de Investigación Clínica de Aragón; CEIC Hospital Clínic i Provincial de Barcelona, Agencia de Ensayos Clínicos – Servicio de Farmacia; CEIC Hospital de Girona “Dr. Josep Trueta”; CEIC Autonómico de Ensayos Clinicos de Andalucía); Sweden (Regionala etikprövningsnämnden); Switzerland (Ethikkommission beider Basel, EKBB; Kantonale Ethikkommission Bern KEK); USA (University of Arkansas for Medical Sciences Institutional Review Board; Thomas Jefferson University Office of Human Research Institutional Review Board; Fox Chase Cancer Center Institutional Review Board; Committee on Research Involving Human Subjects; Schulman Associates IRB; Institutional Review Board, Methodist Hospital, Omaha; UT Health Science Center Institutional Review Board; Dana-Farber Cancer Institute Institutional Review Board; University of Texas MD Anderson Cancer Center; Ingalls Memorial Hospital; Memorial Healthcare System – Western Institutional Review Board).

### Patients

Eligible patients must be at least 18 years of age and have histologically or cytologically confirmed squamous cell carcinoma of the oral cavity, oropharynx, hypopharynx or larynx, which has recurred/metastasised and is not amenable for salvage surgery or radiotherapy. Patients are required to have documented progressive disease (PD) based on investigator assessment according to Response Evaluation Criteria in Solid Tumors (RECIST) following receipt of at least two cycles of cisplatin or carboplatin administered for R/M disease. Patients must have measurable disease according to RECIST Version 1.1 and an ECOG performance status of 0 or 1 at the time of randomisation. Main exclusion criteria include PD within 3 months of completion of curatively intended treatment of LA or metastatic HNSCC, primary tumour site of the nasopharynx (of any histology), sinuses and/or salivary glands, any other than one previous platinum-based systemic regimen given for R/M disease, prior treatment with EGFR-targeted small molecules, and pregnancy or breastfeeding.

### Efficacy assessments

Progression-free survival, the primary endpoint, is defined as the time from the date of randomisation to the date of progression or to the date of death, whichever occurs first. Computed tomography scans or magnetic resonance imaging will be performed at baseline, every 6 weeks during the first 24 weeks after randomisation, and every 8 weeks thereafter. Disease progression will be evaluated according to RECIST Version 1.1 by independent central review. Overall survival, the key secondary endpoint, is defined as the time from the date of randomisation to the date of death (regardless of the cause of death). Other efficacy endpoints include ORR, defined as CR or partial response determined by RECIST Version 1.1, and tumour shrinkage, defined as the maximum decrease in the sum of the longest diameters of the target lesions.

### Safety assessments

Safety endpoints include the overall incidence and intensity of AEs, e.g. gastrointestinal events (vomiting, nausea, diarrhoea), skin reactions (rash, acne) and change from baseline for all laboratory tests. The incidence and intensity of AEs will be graded according to United States National Cancer Institute Common Terminology Criteria for Adverse Events Version 3.0.

### HRQoL assessment

Health-related quality of life will be assessed using questionnaires by the European Organisation for Research and Treatment of Cancer Quality of Life Questionnaire (EORTC QLQ-C30) and the EORTC Head and Neck cancer-specific supplementary module (EORTC QLQ-H&N35). The main analysis of HRQoL questionnaires will focus on the following scales: Pain scale (composite of items 31–34 of the EORTC QLQ-H&N35); swallowing scale (composite of items 35–38 of the EORTC QLQ-H&N35); global health status/QoL scale (composite of items 29 and 30 of the EORTC QLQ-C30). Health-related quality of life data will also be collected using the EQ-5D questionnaire and will be analysed descriptively.

### Biomarker assessment

Participation in the biomarker part of the study is voluntary and not a prerequisite for participation in the trial. A separate informed consent to allow for biomarker analyses must be given in accordance with local ethical and regulatory requirements. Pharmacodynamic biomarker analyses will be based on archival tumour tissue and serum samples. For serum-derived biomarkers, blood samples will be taken from consented patients before the start of treatment (at Visit 2) and an evaluation of the VeriStrat proteomic signature will be performed. For tumour tissue-derived biomarkers, archival tumour tissue will be analysed for the presence of p16 by immunohistochemistry. Additional exploratory biomarker analyses are planned, including determination of ErbB ligands, ErbB receptor expression, EGFR mutation status and EGFR downstream signalling markers.

### Statistical analyses

#### Outcome analyses

Progression-free survival will be analysed using a stratified log-rank test with baseline ECOG performance score and prior use of EGFR-targeted antibodies for R/M HNSCC being the stratification factors. The Kaplan-Meier method will be used to summarise PFS times for each treatment group and the stratified Cox proportional hazards model will be used to determine the hazard ratio between the two treatment groups. OS will be analysed, similarly to PFS, using the stratified logrank test, the Kaplan-Meier method and the Stratified Cox Proportional Hazards model.

### Trial status

**Figure 2:**
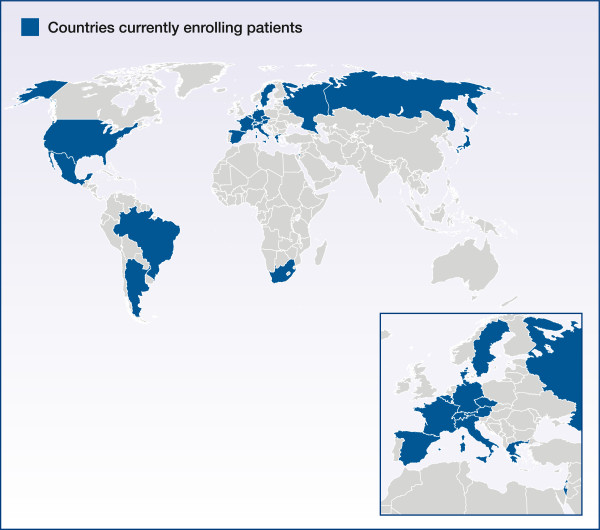
Trial countries.

The trial was initiated in January 2012 and is currently recruiting patients in Argentina, Austria, Belgium, Brazil, Czech Republic, Denmark, France, Germany, Greece, Israel, Italy, Japan, Mexico, Russia, South Africa, Spain, Sweden, Switzerland and the USA (Figure 2).

## Discussion

A detailed account of the LUX-Head & Neck 1 trial has been provided here to increase awareness of the study and provide a detailed rationale for why this study is being performed. Results are anticipated to demonstrate improved efficacy of afatinib compared with methotrexate in platinum-failure HNSCC patients and expand second-line treatment options in this setting to meet the current unmet medical need of these patients. This is one of two Phase III studies of afatinib in HNSCC, with LUX-Head & Neck 2 (NCT01345669) assessing afatinib in the adjuvant setting in unresected, intermediate-to-high risk LA HNSCC patients; this trial is also currently recruiting patients. Afatinib has demonstrated comparable clinical activity to cetuximab in a proof-of-concept study in the second-line treatment of R/M HNSCC [48,49], as well as antitumour activity in preclinical models [46,47]. Afatinib has also shown promising clinical efficacy in NSCLC and it is expected that activity of this nature will be mirrored in the treatment of R/M HNSCC.

## Competing interests

JPHM is an advisory board member for Boehringer Ingelheim, Merck Serono and Symphogen. LFL is a consultant for Boehringer Ingelheim, Bristol-Myers Squibb, GlaxoSmithKline, Eli Lilly, Merck Serono, Amgen, Debiopharm and VentiRX. She has received research funding from Boehringer Ingelheim, Eisai, Exelixis, Eli Lilly, Merck Serono, Amgen and Pfizer, and has received travel reimbursement for medical meeting attendance from Merck Serono and Debiopharm. RIH is an unpaid consultant and has provided research support for Boehringer Ingelheim; he is also a consultant for AstraZeneca and Exelixis. EEWC is the overall Steering Committee Chair for the LUX-Head & Neck 1 and LUX-Head & Neck 2 studies without compensation. MT has received honoraria from Merck Serono and Bristol-Myers Squibb, and has received research funding from Boehringer Ingelheim, Eisai and Yakult Honsya.

## Authors’ contributions

JPHM is the international study coordinator, was involved in the writing, conception and design of the study protocol, and was responsible for approving the final protocol. LFL participated in study design and coordination. RIH was involved in study design and coordination and study protocol conception and design. MT participated in study design and coordination. EEWC participated in study design. All authors are members of the LUX-Head & Neck 1 Publication Steering Committee, were fully responsible for all content and editorial decisions, and were involved at all stages of manuscript development. All authors read and approved the final manuscript.

## Pre-publication history

The pre-publication history for this paper can be accessed here:

http://www.biomedcentral.com/1471-2407/14/473/prepub
